# High-Density Genetic Map Construction and Stem Total Polysaccharide Content-Related QTL Exploration for Chinese Endemic *Dendrobium* (Orchidaceae)

**DOI:** 10.3389/fpls.2018.00398

**Published:** 2018-03-27

**Authors:** Jiangjie Lu, Yuyang Liu, Jing Xu, Ziwei Mei, Yujun Shi, Pengli Liu, Jianbo He, Xiaotong Wang, Yijun Meng, Shangguo Feng, Chenjia Shen, Huizhong Wang

**Affiliations:** ^1^College of Life and Environmental Sciences, Hangzhou Normal University, Hangzhou, China; ^2^Zhejiang Provincial Key Laboratory for Genetic Improvement and Quality Control of Medicinal Plants, Hangzhou Normal University, Hangzhou, China; ^3^State Key Laboratory of Systematic and Evolutionary Botany, Institute of Botany, Chinese Academy of Sciences, Beijing, China; ^4^Center of Rare Plant Medicine Research of Zhejiang Province, Wuyi, China; ^5^Zhejiang ShouXianGu Pharmaceutical Co. Ltd., Wuyi, China; ^6^College of Pharmaceutical Science, Zhejiang Chinese Medical University, Hangzhou, China; ^7^School of Foreign Languages, Zhejiang Gongshang University, Hangzhou, China; ^8^Soybean Research Institute, Nanjing Agricultural University, Nanjing, China

**Keywords:** *Dendrobium*, Genetic linkage map, Polysaccharides, Quantitative trait locus (QTL), SNP

## Abstract

Plants of the *Dendrobium* genus are orchids with not only ornamental value but also high medicinal value. To understand the genetic basis of variations in active ingredients of the stem total polysaccharide contents (STPCs) among different *Dendrobium* species, it is of paramount importance to understand the mechanism of STPC formation and identify genes affecting its process at the whole genome level. Here, we report the first high-density single-nucleotide polymorphism (SNP) integrated genetic map with a good genome coverage of *Dendrobium*. The specific-locus amplified fragment sequencing (SLAF-seq) technology led to identification of 7,013,400 SNPs from 1,503,626 high-quality SLAF markers from two parents (*Dendrobium moniliforme* ♀ × *Dendrobium officinale* ♂) and their interspecific F_1_ hybrid population. The final genetic map contained 8, 573 SLAF markers, covering 19 linkage groups (LGs). This genetic map spanned a length of 2,737.49 cM, where the average distance between markers is 0.32 cM. In total, 5 quantitative trait loci (QTL) related to STPC were identified, 3 of which have candidate genes within the confidence intervals of these stable QTLs based on the *D. officinale* genome sequence. This study will build a foundation up for the mapping of other medicinal-related traits and provide an important reference for the molecular breeding of these Chinese herb.

## Introduction

Species of the orchid genus *Dendrobium* are among the herbs that have been used in traditional Chinese medicine in China and many other Asian countries for centuries. There are 74 species of *Dendrobium* mainly distributed in the southern Tsinling Mountains of China (Tsi et al., 1999). The desiccated and spring-shape rolled stems of *D. officinale, D. nobile*, *D. fimbriatum*, and *D. chrysotoxum* are used for the treatment of throat inflammation, fever, and gastritis, or for the immune stimulation and anti-tumor properties (Bulpitt et al., 2007; Committee, 2015). Modern pharmacological studies have shown that several active ingredients of polysaccharides, alkaloids, flavones, phenols, and benzyl compounds in *Dendrobium* have important medicinal effects (Tian et al., 2015; Huang et al., 2016). As the main active ingredients in the stem, total water-soluble polysaccharides, have been demonstrated to show prominent bioactivities (Luo et al., 2008; Meng et al., 2013). Accordingly, breeding cultivars with a higher stem total polysaccharide content (STPC) is one of the main objectives in the *Dendrobium* industry.

Many studies have attempted to determine the genetic basis of STPC. The genes of *CELLULOSE SYNTHASE-LIKE A* (*CSLA*) (He et al., 2015) and GDP-mannose pyrophosphorylase (*DoGMP1*) (He et al., 2017) have been reported to play important roles in the biosynthesis of mannan polysaccharides and in abiotic stress responses in *D. officinale*. Using high-throughput sequencing technology, 170 differentially expressed gene (DEGs) belonged to 28 the glycosyltransferase genes (GTs) were identified from transcriptome databases of juvenile and adult *D. officinale* cDNA libraries (Zhang J.X. et al., 2016), and 280 glycosyltransferase genes have been identified in the flowers, leaves, stems, and roots transcriptomes of *D. officinale*, respectively (Shen et al., 2017). These genes were speculated to be indirectly associated with STPC in *D. officinale* due to their differential gene expression pattern as validated by real-time PCR and sequence homologies to Arabidopsis genes with conformed functions. To date, however, the precise genetic basis of STPC in *Dendrobium* has not been determined.

Dendrobe STPCs are among the quantitative traits in *Dendrobium*, including a widespread group of related major and minor quantitative trait loci (QTL), which containing the genes and enzymes participate in glycan and glycoside biosynthesis (Lao et al., 2014). Mapping the STPC associated QTLs in the *Dendrobium* genome will enable us determine the genetic basis of these traits and carry out marker-assisted selection (MAS) of STPC in this medicinal crop. Nevertheless, there are difficulties in constructing mapping populations for *Dendrobium* due to self-incompatibility (Tremblay et al., 2004) and the low levels of self-reproduction (Wang et al., 1999). In our previous studies, we constructed framework genetic maps for five *Dendrobium* species, namely, *D. officinale, D. hercoglossum, D. nobile, D. moniliforme* and *D. adumcum* (Xue et al., 2010; Lu et al., 2012a,b; Feng et al., 2013). However, these maps showed a low-density coverage, consisting of hundreds of RAPD (651), SRAP (485), ISSR (154), and SSR (142) markers, leading them difficult to be used in the fine mapping of important traits. To the best of our knowledge, neither complete genetic map, nor comprehensive QTL mapping has previously been reported for any *Dendrobium* species.

Recent advances in next-generation sequencing (NGS) technologies have provided effective platforms for high-quality SNP marker detection without a reference genome (Perkel, 2008; Davey et al., 2011). For example, the sequencing of restriction-site associated DNA (RAD-seq) (Baird et al., 2008) and specific-locus amplified fragment sequencing (SLAF-seq) (Sun et al., 2013) are the two most common NGS-based technologies, which provide the massive SNPs of mapping populations for high-density map construction. SLAF has been used in orchardgrass (Zhao et al., 2016), cotton (Zhang Z. et al., 2016), *Salix matsudana* (Zhang J. et al., 2016), watermelon (Shang et al., 2016), mango (Luo et al., 2016), and *Prunus mume* (Zhang et al., 2015) with high resolution for large-scale genotyping, and has been demonstrated to be an ideal tool in QTL mapping and gene discovery studies. The main purpose of the present study was to improve the QTL mapping resolution and expand the knowledge of STPCs in *Dendrobium*. High-density linkage maps were constructed through SLAF-seq using a mapping population of inter-specific crosses (*D. moniliforme* ♀ × *D. officinale* ♂) firstly, followed by examination of the genetic bases underlying STPC. The availability of such a high-density linkage map will provide a much-wanted tool to identify smaller genomic regions for target traits associated alleles, and to analyze the allelic differences between *Dendrobium* wild species, thereby broadening the genetic bases of *Dendrobium* cultivars.

## Materials and methods

### Plant materials and total DNA extraction

**Figure 1:**
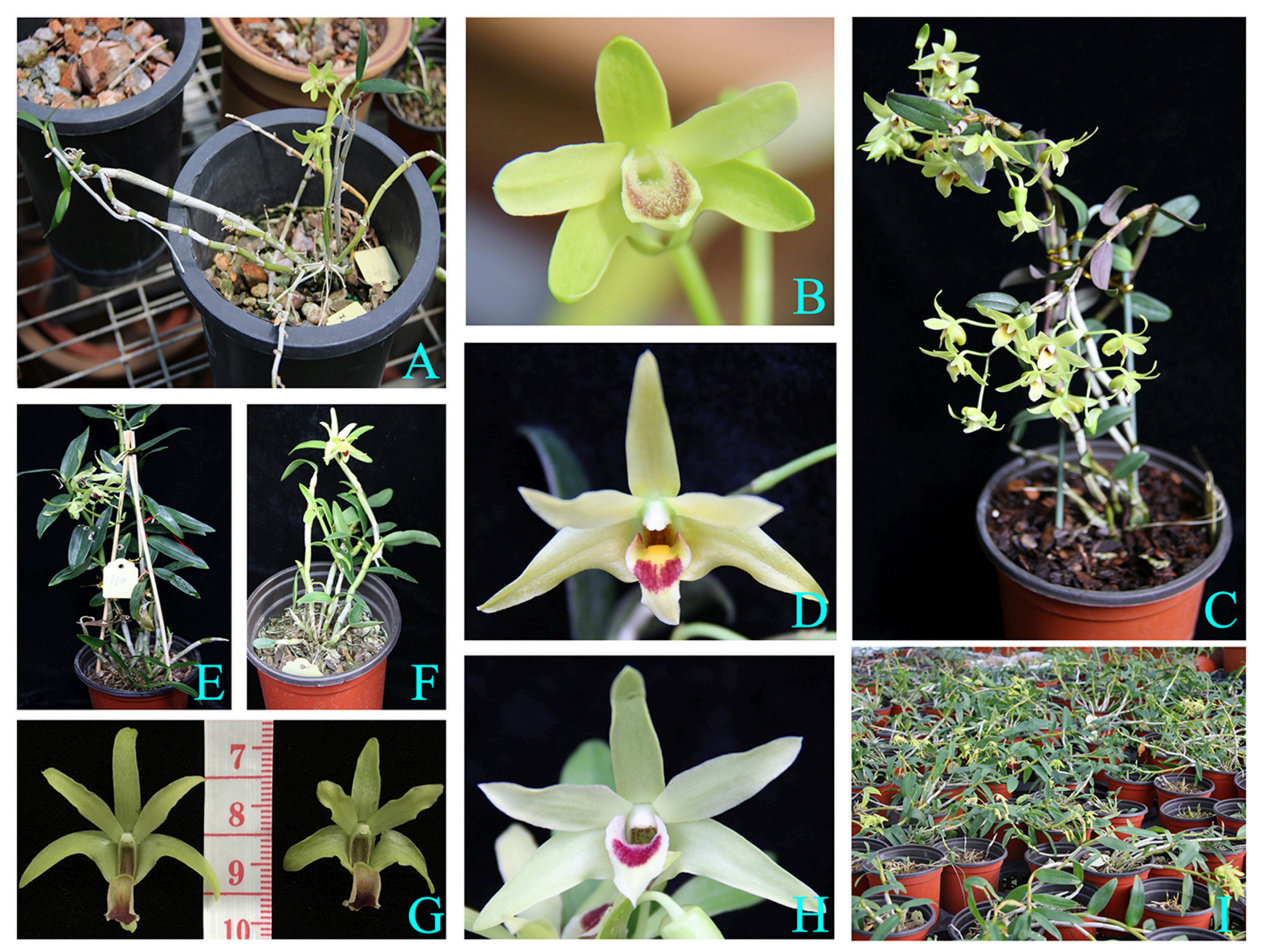
The phenotypic characteristics of the parents and the hybridization F_1_ population. **(A)** The female parent *D. moniliforme*. **(B)** The flower of female parent. **(C)** The male parent *D. officinale*. **(D)** The flower of male parent. **(E)** The hybridization F_1_ individual 110. **(F)** The hybridization F_1_ individual 98. **(G)** The flower of hybridization F_1_ individual 110 (left) and 98 (right). **(H)** The flower of hybridization F_1_ individual 56. **(I)** The profile of hybridization F_1_ population.

The female parent *D. moniliforme* (PE 00293907) was derived from a wild population in LuXi county of YunNan province in China; whereas, the male parent *D. officinale* (PE 00293966) was an F_4_ generation artificially selfing dendrobe strain named XIANHU #1 (Non-staple crop approval variety of Zhejiang Province, China. Certificate No: 2008003) provided by the Zhejiang ShouXianGu Pharmaceutical Co. Ltd., Zhejiang, China. The female parent was identified with ITS2, the sequence (MG940864) is 99% identity with ITS2 of *D. moniliforme* (KP159303, KF143489, HQ114146, and KJ210480). Two flowering plants before hybridization and the population individuals are shown in Figure 1. The parents were collected and transplanted to the greenhouse of Hangzhou Normal University (120°19′E, 30°26′N), where they have been used for hybridization since 2013. Cross-pollination seeds were germinated in a lightly moistened potting mix at about 25°C, in order to ensure the survival rate of seedlings at the transplant stage. After 3 months, the strong and healthy seedlings were transplanted into a nursery garden, and grow healthily in the environment of temperature at about 25°C, humidity at about 60%, in order to avoid the effect of environmental heterogeneity to the STPS. Finally, a total of 111 two-years old interspecies hybrid individuals with at least 150 g biomass were randomly selected for genetic linkage map construction and polysaccharides content determination.

The total genomic DNA of mapping population was extracted from fresh leaf with modified CTAB method described previously (Wang et al., 2009). The concentration was determined by NanoDrop™ 1000 spectrophotometer (Thermo Fisher Scientific, Wilmington, USA) and the purity was checked with 1% agarose gel electrophoresis for all the DNA samples.

### Library construction and sequencing

In marker discovery experiments, the reference genome of the GREEN selfing strain of *D. officinale* (Yan et al., 2015) was used, where the number of markers was predicted by simulating *in silico* under different enzymes combination. After the pilot experiment, an SLAF library was conducted. Next, *Hae* III and *Hpy* 166II (New England Biolabs, USA) were used in the genomic DNA digestion. Thereafter, an A nucleotide overhang was added to the digested fragments by Klenow Fragment (3′ → 5′ exo^−^) (New England Biolabs) and dATP. Subsequently, the A-tailed fragments were ligated with PAGE-purified Duplex tag-labeled sequencing adapters. PCR was performed using dNTP, PCR primer (Forward: 5′-AATGATACGGCGACCACCGA-3′; Reverse: 5′-CAAGCAGAAGACGGCATACG-3′), diluted restriction-ligation DNA samples, and Q5® High-Fidelity DNA Polymerase. After purifying with Agencourt AMPure XP beads (Beckman Coulter, High Wycombe, UK), the PCR products were separated by 2% agarose gel electrophoresis. Excised fragments with size from 314 to 394 bp (with indexes and adaptors) were purified using a QIAquick gel extraction kit (Qiagen, Hilden, Germany). All the purified products were then diluted and sequenced using an Illumina HiSeq 2500 sequencer (Illumina, Inc.; San Diego, CA, USA) with the HiSeq SBS Kit v4 (paired-end 125-bp reads) according to the manufacturer's recommendations. Based on the polymorphic analysis of two parents and the reported genome size, balance of genotyping accuracy and sequencing cost, the expected sequencing depth of all sample is 20-fold.

### SLAF marker identification and genotyping

SLAF marker identification and genotyping were performed using procedures described by Sun et al. (2013). Briefly, after filtering the low-quality reads (quality score <20^*e*^) and sorting the raw reads according to duplex barcode sequences of each progeny, high-quality clean reads were mapped onto the *D. officinale* GREEN genome sequence (Yan et al., 2015) using SOAP software for every sample (Li et al., 2009). Same-position mapped sequences with identity ≥95% were defined as a single SLAF tag (Tao et al., 2017). Filtered out the SLAF tags with more than three SNPs initially after the SNPs detection of each SLAF tag between parents. For each SLAF tag, the alleles were defined according to parental reads with sequence depth >150-fold, whereas a sequence depth >40-fold was used to define alleles for each offspring read. *D. moniliforme* and *D. officinale* are diploid species, so one SLAF locus contains at most four genotypes, and therefore, SLAFs with more than four alleles were discarded as repetitive. Correspondingly, SLAFs with two to four alleles were identified as potential polymorphic SLAFs. The marker code of the polymorphic SLAFs were analyzed based on the cross pollinators (CP) population type, which consist of eight segregation types (ab × cd, ef × eg, ab × cc, cc × ab, hk × hk, lm × ll, nn × np, and aa × bb). The ab × cd type represents the two alleles in both parents that are different for the markers.

A Bayesian approach was applied to further ensure the genotyping quality for genotype scoring (Zhang et al., 2015). Initially, the posteriori conditional probability was calculated using the coverage of a number of SNPs and haplotype for each SLAF. The probability was then translated into a genotyping quality score, which was used to select qualified markers for subsequent analysis during the dynamic optimization process (Sun et al., 2013). The process will not be stopped until the average genotyping quality scores reached the cut-off value for all SLAFs. Subsequently, high-quality genetic mapping SLAF markers were filtered under the criteria of average sequence depths >20-fold and >8-fold for parents and progeny, respectively. Markers with more than 5% missing data were filtered out. The chi-square test was performed to examine the segregation distortion, and the significant segregation distortion markers (*P* < 0.05) were initially excluded from map construction.

### Linkage analysis and map construction

After partitioning the filtered marker into linkage groups (LGs) based on their locations on the GREEN genome, we used the MLOD scores >5 between markers to confirm the robustness of markers for each LG. The HighMap strategy was utilized to order the SLAF markers and correct genotyping errors within LGs, in order to ensure the efficient construction of a high-density and high-quality map (Liu D.Y. et al., 2014). The LGs were constructed as follows: primary linkage phases were inferred from the recombinant frequencies and LOD scores, and the error correction strategy of SMOOTH (Van Os et al., 2005) was then conducted according to the parental contribution of genotypes, and missing genotypes were imputed with a k-nearest neighbor algorithm (Huang et al., 2012). In order to conduct an iterative process of marker ordering, three strategies of enhanced Gibbs sampling, spatial sampling, and simulated annealing algorithms were applied together (Jansen et al., 2001; Van Ooijen, 2011). The map density was further increased with skewed markers by applying the multipoint method of maximum likelihood. These processes were performed iteratively to ensure the accuracy of maker order and map distances. After 3–4 cycles, we can perform the next round of map building once a stable map order was obtained. All the currently unmapped markers were selected and added to the previous sample with decreased sample radius. The Kosambi mapping function (Kosambi, 1943) was used to estimate the map distances.

### Stem total polysaccharides contents determination and QTL analysis

The phenol-sulfuric acid method recommended in the *Pharmacopeia of The People's Republic of China* (first volume of the 2015 Edition) was applied to determine the STPC of 2 years old stems with some modification. The STPC determination were repeated three times for two parents and 111 F1 individuals, with every repeat used dry powder (sifted with 50 mesh powder sifter) from 30 g fresh stems of each individual. In order to guarantee the accuracy of the absorbance value, we increased the amount of powder used from 0.3 to 0.45 g, so that the absorbance value was almost greater than 0.2 at 488 nm. The purpose of the typical 1 h cold storage in the standard protocol is to facilitate complete alcohol precipitation of polysaccharides, and therefore we prolonged this stage to more than 1 h, or even precipitated overnight. The standard 4000 rpm centrifugation for 20 min was changed to a 7,000 rpm centrifugation for 30 min at 5°C, in order to enhance the effect of polysaccharide precipitation. Redistilled phenol was used instead of normal phenol, because higher purity phenol is considered more effective in producing the golden-yellow color of the sulfuric acid break down products of polysaccharides.

The SPSS 18.0 was used to analyze the STPC phenotypic data. The interval mapping (IM) method in MapQTL® 6.0 (Van Ooijen, 2009) software was used for the QTL mapping. We employed a modified sliding window of 1-cM to scan the genome. The maximum LOD score along the interval was considered as the position of the QTL, and the confidence interval was defined as the region in the LOD score greater than the threshold. Initially, both the genome and chromosomes wide LOD threshold from 1,000-permutation test at significance levels of 0.05 and 0.1 was used separately, but these two thresholds seemed too stringent for the present study and no STPC related QTLs were found. In consideration of the statistical significance for STPC variation in our population, therefore, a less stringent LOD threshold was determined with a confidence of 0.75, in order to get the reasonable QTLs number of STPC under relative stringent LOD.

### QTL associated markers annotation information extraction

All the STPC associated markers were realigned back to the reference genome of *D. officinale* GREEN (Yan et al., 2015), their accurate and physical locations to the scaffolds and the Gene ID were determined. All the corresponding annotation information, such as COG class and annotation, functional annotation of GO, KEGG, Swissprot and Nr, were also extract from the original reference genome annotation, in order to explore the gene function of STPC associated markers.

## Results

### SLAF-seq data analysis of 111 F_1_ individuals and two parents

In this study, based on the results of an SLAF pilot experiment, we initially constructed an SLAF-seq library for *Dendrobium* using 111 F_1_ individuals and their parents. A total of 616,883,449 pair-end reads were obtained, 94.15% bases had a high quality Q score > 30, and the average GC content was 38.96%. The number of reads for male and female parents was 22,761,289 and 22,997,150, respectively. The average clean read numbers were up to 5,145,270 for the F_1_ population (Table 1).

**Table 1:** SLAF-seq data summary for dendrobium F_1_ population.

**TOTAL READS**	
No. of clean reads	616,883,449
No. of clean reads of male parent	22,761,289
No. of clean reads of female parent	22,997,150
Average no. of clean reads of F_1_ individual	5,145,270
Average Q30 percentage (%)	94.15
Average GC percentage (%)	38.96
**HIGH-QUALITY SLAFS**	
No. of SLAFs	1,503,626
No. of polymorphic SLAFs	946,508
Average SLAF depth (X)	304.00
Average depth in parents (X)	163.64
Average depth in individuals (X)	41.91
**HIGH-QUALITY SNP MARKERS**	
Total No. of SNPs	1,932,745
No. of SNPs can genotyping	697,716
No. of SNPs can used for mapping	398,083
No. of high-quality SNPs	8,836

Based on the sequence similarity, all reads were clustered into SLAFs. After correcting and discarding the low-depth SLAF tags, a total of 1,503,626 high-quality SLAFs were identified, of which 630,555 and 737,197 were detected in the male and female parents, respectively. The coverage of SLAFs for paternal and maternal genomes was 24.48- and 23.04-fold, respectively. For the F_1_ population progeny, 327,332 to 494,382 SLAFs (average, 404,616) were generated, and the average SLAFs coverage was 9.35-fold, ranging from 5.43- to 16.26-fold (Supplementary Figure 1).

### SLAF detection and genotype definition

**Figure 2:**
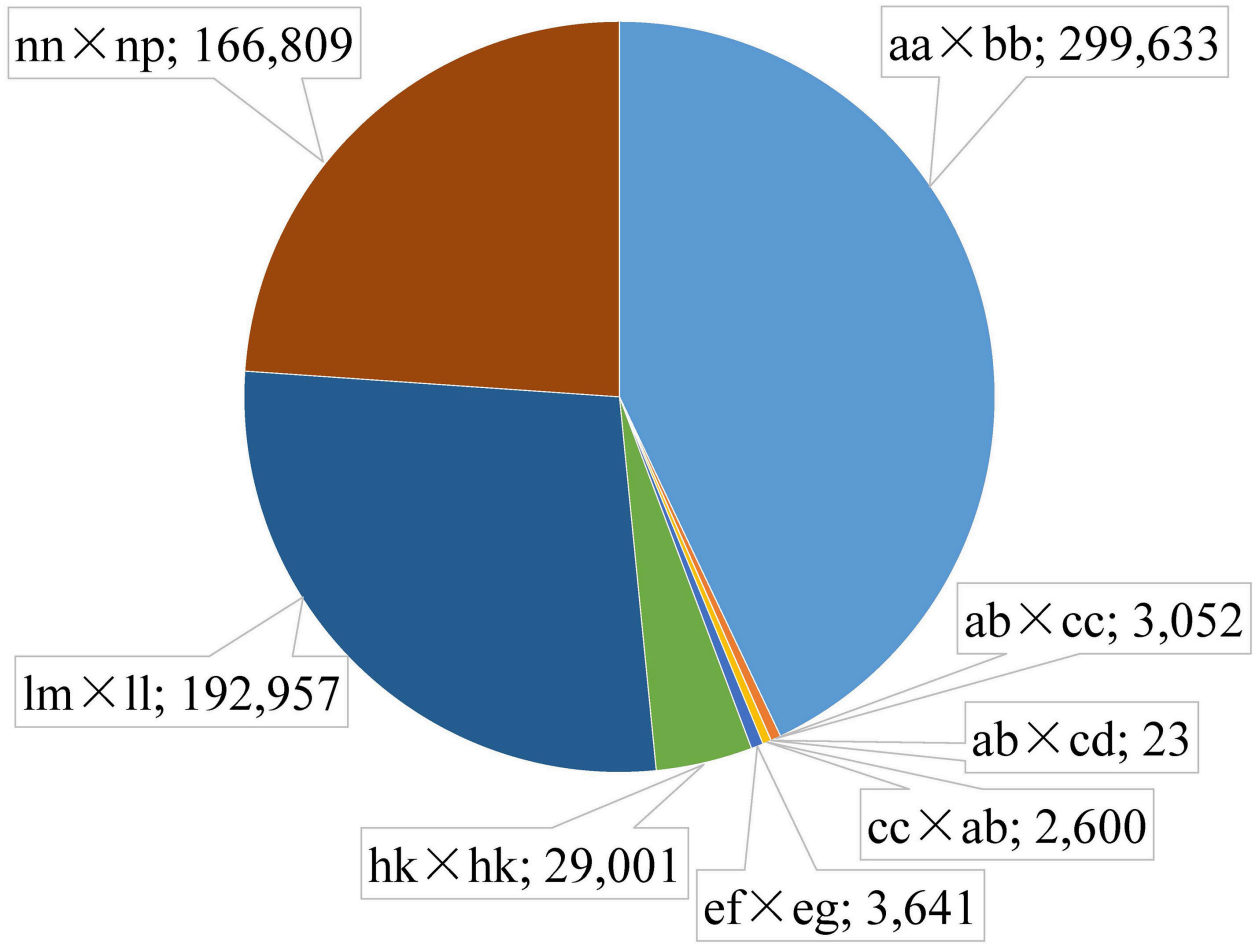
Distribution of SLAF markers in eight segregation patterns. The segregation patter and number is separated by a semicolon.

Among the 1,503,626 obtained high-quality SLAFs, the polymorphism percentage is 62.95% with 946,508 polymorphic SLAFs (Table 1). The 946,508 polymorphic SLAFs harbored a total of 1,932,745 SNPs, as one SLAF generally harbored one to three SNPs. Among the 1,932,745 SNPs, 697,716 were identified as being polymorphic within population with the polymorphic rate at 36.10%. All the polymorphic SNPs were genotyped with eight segregated patterns: ab × cd, ef × eg, ab × cc, cc × ab, hk × hk, lm × ll, nn × np, and aa × bb (Figure 2). As the segregation pattern of aa × bb was not applicable to the F_1_ population, a total of 398,083 SNPs from the other seven segregation patterns were used for map construction. In order to further enhance the genetic map accuracy, only SNPs with greater than 95% integrity and average sequence depths ≥ 20-fold in the parents and ≥ 8-fold in the F_1_ individuals were used for map construction. Partial segregation markers were filtered at the *P* < 0.05 level. Finally, 8,836 of the 398,083 markers were winnowed out for linkage map construction (Table 2). Among these 8,836 markers, average sequencing depths were 158.86-fold for the male parent and 168.42-fold for the female parent, and from 18.7 to 96.45 (with an average of 41.91-fold) for each progeny.

**Table 2:** Statistics for segregation types of the SLAF markers selected for the linkage map construction.

**Type**	**SNP number**	**Percentage**
ef × eg	18	0.20%
hk × hk	80	0.91%
lm × ll	4,509	51.03%
nn × np	4,229	47.86%
Total	8,836	100%

### High-density linkage map construction

**Figure 3:**
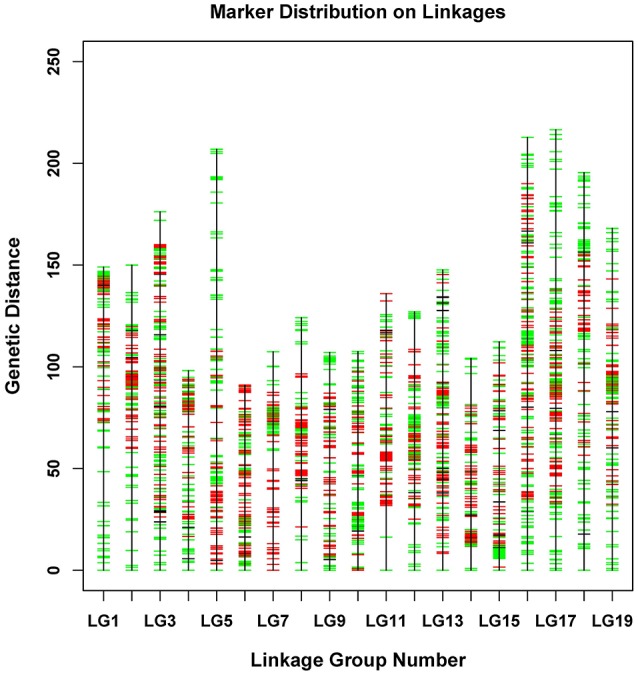
Distribution of SNPs on 19 linkage groups. The x-axis represents linkage group number and the y-axis indicates genetic distance (centiMorgan as unit). SNPs positions are marked in red (from male parent), green (from female parent), and black (from both parents).

The newly developed 8,836 SNPs were used to construct the genetic linkage map for *Dendrobium*. After linkage analysis, the genetic map with 19 linkage groups was constructed by 8,573 markers (Table 3, Figure 3, and Supplementary Table 1). This final map was 2,737.49 cM in length, with mean LG length was 144.08 cM and an average inter-marker distance of 0.32 cM. The markers number mapped to each linkage group ranged from 322 in LG4 to 661 markers in LG13, with an average of 451 per LG. The longest LG (216.52 cM) was LG17, which contained 647 markers, and the shortest LG was LG6, which contained only 398 markers with a length of 91.05 cM. The “Gap <5 cM” value of the 19 linkage groups ranged from 96.29 to 99.75% (average, 98.94%) (Table 3). The maximum gap was 16.28 cM in LG11 between the Marker 489436 and Marker 265690 (Supplementary Table 2). We also construct the sex-specific maps in our study. The male map contained 4,527 markers with mean of 241 markers per LG, and spanned 2,367.29 cM with the average inter-marker distances was 0.52, whereas the female map contained 4,144 markers with an average of 218 markers per LG, and spanned 3,163.23 cM with the average inter-marker distances was 0.76 cM. The total genetic map length of the female is considerably larger than that of the male, and 15 LGs in the female map have larger genetic distance. Some significant differences were also be observed in some LGs, for example, the lengths of LG7, LG9, LG10, and LG14 in the male map are larger than those in the female map (Supplementary Table 3).

**Table 3:** Basic characteristics of *Dendrobium* linkage groups.

**LG ID**	**Total marker**	**Total distance (cM)**	**Average distance (cM)**	**Max gap (cM)**	Gap < 5 cM^*^ (%)	**Partial segregation SLAF**
1	395	148.99	0.38	15.08	98.73	0
2	557	149.96	0.27	13.51	98.56	0
3	519	176.16	0.34	11.94	99.42	3
4	322	98.16	0.30	6.23	99.69	0
5	351	206.97	0.59	15.68	96.29	0
6	398	91.05	0.23	5.03	99.75	5
7	338	107.48	0.32	9.19	98.52	0
8	377	124.19	0.33	14.79	98.67	0
9	349	107.15	0.31	7.07	99.43	1
10	375	107.56	0.29	6.30	98.93	6
11	401	135.91	0.34	16.28	98.50	7
12	501	127.03	0.25	15.23	98.80	0
13	661	147.60	0.22	8.32	99.55	3
14	435	104.22	0.24	15.23	99.31	2
15	330	112.33	0.34	6.33	99.39	0
16	633	212.72	0.34	8.23	99.68	0
17	647	216.52	0.33	13.48	99.07	1
18	340	195.44	0.57	10.68	97.05	21
19	644	168.05	0.26	7.67	99.07	1
Total	8,573	2,737.49	0.32	16.28	98.94	50

* *The “Gap <5 cM” value means the percentages of gaps in which the distance between adjacent markers is smaller than 5 cM*.

### Fine mapping of loci conferring STPC

According to the standards in the *Pharmacopeia of The People's Republic of China* (first volume of the 2015 Edition), the STPC is the primary quality character for DENDROBII OFFICINALIS CAULIS, and the minimum content is 25% (Calculate with C_6_H_12_O_6_). From the basic statistics for the STPC listed in Supplementary Table 4, we found broad phenotypic variation of the STPC among the 111 individuals, ranging from 5.28 to 67.06, 6.48 to 68.32, and 6.21 to 67.58 for three repeats, with average from 5.99 to 67.65%. The ANOVA indicated no obvious differences for three repeats (mean square is 11.51), but the significant genotypic variation among the 111 individuals with mean square is 526.14 (Supplementary Figure 2).

For 19 LGs of our high-density *Dendrobium* genetic map, when the significance level of 0.25 in permutation test were selected, the LOD thresholds of all chromosomes were higher than 2, and two QTLs were detected in LG2 with LOD threshold at 2.3, one QTL in LG11 with LOD threshold at 2.4, and two QTLs in LG15 with LOD threshold at 2.3 (Supplementary Table 5). The phenotypic variability explained by each QTL ranged from 9.20 to 11.80% (average, 10.20%). Two QTLs of STPC-1 and STPC-2 were located with confidence intervals at 78.821–79.644 and 90.349 cM on LG2, showing a percentage of phenotypic variance explained (PVE) of 9.70 and 9.20% (Supplementary Figure 3). The LG11 harbored only one QTL, namely, STPC-3 at 85.984 cM, with PVE values of 11.80% (Supplementary Figure 4). STPC-4 and STPC-5 were found on LG15 in the regions of 87.955–88.241 cM and 95.533–95.899 cM, respectively, with corresponding PVE values of 9.40 and 10.90% (Supplementary Figure 5).

### Annotated genes within the candidate region

There are a total of 19 SNP markers in the regions of the 5 identified STPC-associated QTLs. Among these, STPC-3 is single marker-associated QTLs, and STPC-1 has the largest marker number with 8 SNPs. On the basis of annotation of the reference genome of GREEN selfing strain of *D. officinale* (Yan et al., 2015), we determined that 8 of the 19 STPC-associated SNPs were located in 4 genes. Two of 4 STPC-associated genes were mapped to reference canonical pathways in KEGG, they encoded putative RNA polymerase II-associated factor 1 (K15174) and interleukin-1 receptor-associated kinase 4 (K04733) (Supplementary Table 5).

GO term enrichment analysis showed that 3 of these genes were enriched with a total number of 16 GO terms in various biological processes, cellular components, and molecular functions. The gene Dendrobium_GLEAN_10032348 in QTL STPC-1 has three GO terms of cellular component: mitochondrion (GO: 0005739); biological process: protein targeting to vacuole (GO: 0006623) and vesicle-mediated transport (GO: 0016192). The gene Dendrobium_GLEAN_10047849 in QTL STPC-5 has three GO terms of molecular function: protein serine/threonine kinase activity (GO: 0004674) and ATP binding (GO: 0005524); and biological process: protein phosphorylation (GO: 0006468). In addition, the gene Dendrobium_GLEAN_10120125 has 10 GO terms, including two cellular component: mitochondrion (GO: 0005739) and plastid (GO: 0009536); and eight biological process: mitotic G2 phase (GO: 0000085), photomorphogenesis (GO: 0009640), negative regulation of flower development (GO: 0009910), cullin deneddylation (GO: 0010388), protein ubiquitination (GO: 0016567), histone methylation (GO: 0016571), protein deubiquitination (GO: 0016579), positive regulation of transcription, and DNA-templated (GO: 0045893) (Supplementary Table 5).

## Discussion

### Characteristics of the STPC in the *Dendrobium* plants

Although *Dendrobium* plants are generally diploid, triploid and tetraploid plants are also often found. The basic chromosome number of *Dendrobium* species is *n* = 19 and 20, with a few having *n* = 18 (Jones et al., 1982). Here, we selected *D. officinale* and *D. moniliforme* from the section *Dendrobium* as parental materials, and both species contain the same number of chromosomes (2*n* = 38) (Cheng, 1985). They also have a close genetic relationship based on ISSR molecular analysis, suggesting the potential to generate a viable hybrid between these species (Wang et al., 2009). Furthermore, there is a great degree of phenotypic divergence between these two species, such as in terms of STPC, the shapes and colors of leaf, flower, and labellum (Figure 1). Finally, an F_1_ interspecific hybrid population was created, and 111 seedlings of this population were used for SNP genotyping.

Polysaccharides, including starch and non-starch polysaccharides, are the main constituent of plant biomass and represent an energy source for humans (Sorek et al., 2014). Non-cellulosic polymers, cellulose, and pectic polysaccharides are three main groups of non-starch polysaccharides, which serve not only as the basic elements of the cell wall, but also an energy storage material (Sinha et al., 2011). As a sub-group of non-cellulosic polymers, mannan polysaccharides play an important role in higher plants. They act as structural elements in the cell wall (Schröder et al., 2009), and serve as reserve polysaccharides to feed cells and adjust osmotic potential in the walls of seed endosperm and vacuoles (Meier and Reid, 1982). In addition, polysaccharides of plant and fungal origins broadly manifest antioxidant activity (Wang et al., 2010; Liu Q. et al., 2014; Liu et al., 2017). *Dendrobium* plants are rich in polysaccharides. Their amounts present in mature plants are variable and controlled by both genetic and environmental factors, and STPC is one of the typical quantitative traits. Accordingly, there exist a very widespread group of carbohydrate-active related genes and enzymes, which participate in glycan and glycoside biosynthesis (Lao et al., 2014).

In addition, the polysaccharide contents are even higher in the outstanding *Dendrobium* cultivars cultured under favorable light, temperature, and humidity conditions in farmlands or greenhouses than those of plants growing in the wild (Xing et al., 2013). Thus, in terms of the mass-production of polysaccharides, developing *Dendrobium* cultivars suitable for plantlet culturing in “indoor plant factories” could be a good alternative to harvesting mature *Dendrobium* plants in farmlands. Mapping the STPC-associated QTLs in the *Dendrobium* genome will enable us to gain an understanding the genetic basis and carrying out MAS of STPC in this medicinal crop. As a simple and rapid colorimetric method, phenol-sulfuric acid method is widely used in measurement of total carbohydrates, including mono-, di-, oligo-, and polysaccharides. In our study, following the recommends in the *Pharmacopeia of The People's Republic of China*, this method was applied to determine the STPC of 2-year old stems from *D. officinale* male parent, *D. moniliforme* female parent, and 111 F_1_ individuals. The mean STPC of the male parent *D. officinale* was 42.67%, whereas that of the female parent *D. moniliforme* was 15.06%, and that of the 111 individuals ranged from 5.99 to 67.65%, with mean squares of the two parents and 111 F_1_ individuals of 11.51 and 526.14 (*P* < 0.01), respectively (Supplementary Figure 2 and Supplementary Table 4).

### SLAF-seq strategy and densest genetic map construction of *Dendrobium*

High-density linkage maps are particularly important for QTL mapping of important traits, comparative genomic studies, MAS, and functional gene cloning and identification. *Dendrobium* plants are either epiphytic or lithophytic, and have a long growth cycle during which they produce numerous buds and aerial roots (Ng et al., 2012). These cross-pollinated plants have complex genetic background, highly heterozygous genetic loci, and low self-seed set. These features have contributed to the difficulty in complete genetic map construction of *Dendrobium* species (Wang et al., 1999). To date, there has been relatively limited genetic information available for *Dendrobium* (Teixeira Da Silva et al., 2016). Since 2010, we have used 209 RAPD and 98 SRAP markers to construct the first genetic maps of *D. officinale* and *D. hercoglossum* (Xue et al., 2010). Subsequently, other framework genetic maps of *D. nobile, D. moniliforme* and *D. aduncum* have also been constructed (Lu et al., 2012a,b; Feng et al., 2013). However, these maps showed a low density coverage, consisting of hundreds of RAPD (651), SRAP (485), ISSR (154), and SSR (142) markers, making fine mapping difficulty for important traits in *Dendrobium*.

Although the NGS strategy provides genome-wide SNP identification, NGS data still inevitably suffer from genotyping errors and missing values, particularly when sequencing depths are low and genotypes are highly heterozygous (Bansal et al., 2010; Nielsen et al., 2011; Xu et al., 2012; Wang et al., 2013). Therefore, strict criteria are used during SLAF marker development and genotyping to avoid false-positive markers. When using the SLAF-seq approach, the minimum sequencing depth for each individual is 6-fold (Sun et al., 2013). In our study, SLAF-seq detected 1,503,626 high-quality SLAF in an F_1_ population of *Dendrobium*, 946,508 of which were polymorphic (Table 1), the average Q30 was up to 94.18%, and the average sequencing depths for the male and female parents were 158.86- and 168.42-fold, respectively, and nearly 41.91-fold for each progeny. Finally, 8,836 markers with genotyping quality scores reaching 30 were used for linkage mapping. The markers with significant segregation distortion were excluded, ensuring the quality of the SLAF markers and genotyping accuracy.

The high-density integrated genetic map for *Dendrobium* constructed in the present study spans 2,737.49 cM, and contains 8,573 markers with a resolution of 0.32 cM. The male map contained 4,527 markers and spans 2,367.29 cM, whereas the female map contains 4,144 markers and spans 3,163.23 cM. These genetic maps represent a considerable improvement compared with previous maps (Xue et al., 2010; Lu et al., 2012a,b; Feng et al., 2013), and accordingly provide an important tool for fine mapping of the QTLs for important traits. Approximately 98.94% of the gaps between markers are <5 cM, although we also observed the maximum gap in the integrated map is 16.28 cM in LG 11 (Table 3). For the male and female parent maps, the percentage of gaps <5 cM was 97.47 and 95.47%, respectively (Supplementary Table 3), with maximum gaps of 33.87 cM in LG 18 of the male parent map and 49.8 cM in LG 4 of female parent map. These findings indicates that there may be a higher level of recombination or that markers have not been developed in these regions. Hence, in future, the population size needs to be increased, and other enzymes combination should be used in SLAF library construction. Furthermore, although two parental species have the same chromosome number, the map length was larger in female than in male. Of course, other factors, such as marker number and types, mapping population size, and genotyping accuracy, also affect the genetic length of a linkage map. In order to avoid artificial inflation of map length, we used strict criteria during the process of SLAF marker development and genotyping in this study. So the elongation of the female genetic map is probably attributed to the population size in our views.

### STPC related QTLs analysis utilizing the densest genetic map

In the process of QTL mapping, LOD is one key factor for judging the linkage between two loci, and it is influenced by trait heritability, population size, and density of genetic map. There are no strict rules of LOD cutoff for QTL mapping, although LOD ≥ 3 is the conventional cutoff in classical QTL mapping for crops. In our study, we first run a genome wide permutation test at a significance level of 0.05 in STPC related QTL detection, but there was no significant QTL detected. Thereafter, a less stringent LOD threshold at 0.1 was used to declare suggestive STPC related QTLs, but QTL was still not detected. In order to get the QTLs with the relative stringent LOD, we decreased the LOD threshold gradually. Five STPC related QTLs were revealed when the significance level dropped to 0.25. If we continue drop the significance level to 0.3, the LOD threshold of LG7 was lower than 2. In order to ensure an overall false positive (type 1 error) rate for QTL detection, a typical LOD threshold larger than 2 was suggest (Lander and Botstein, 1989). Therefore, we choice the significance level of 0.25 in permutation test, the LOD thresholds of all chromosomes are higher than 2, and two QTLs were detected in LG2 with LOD threshold at 2.3, one QTL was detected in LG11 with LOD threshold at 2.4, and two QTLs were detected in LG15 with LOD threshold at 2.3.

For the 5 STPC related QTLs detected in our study, the LOD peaks were raged from 2.32 of STPC-1 to 2.66 of STPC-3, with an average of 2.42 (Supplementary Table 5). We attributed the STPC related QTL without high LOD to the lack of larger mapping population and the lower heritability of STPC. Although QTL mapping study is difficult in the slow growth perennial *Dendrobium* species, we first constructed high-density genetic map and localized QTLs using F1 mapping population of 111 individuals with a sufficient amount of biomass. Although the STPC is the primary quality character of STPC required for Dendrobium by the Pharmacopeia of The People's Republic of China (Page 282 of first volume of the 2015 Edition) (Committee, 2015) (Committee, 2015), we'll expand the STPC-related phenotypes to the single chemical components and other agronomic traits with higher heritability in a future study.

### Annotation of STPC related QTLs

Among the solutes and macromolecules in a cell, non-starch polysaccharides are formed by plant cells at certain development stages, typically during periods of intense photosynthetic activity. Thus, when stored, these polysaccharides are temporarily withdrawn from cellular metabolism. Their deposition takes place in the cell wall region, plastids, or in cell vacuoles (Mulimani and Prashanth, 2002). Gene annotation in this study (Supplementary Table 5) identified the vesicle-mediated transport related gene *Dendrobium_GLEAN_10032348* in QTL STPC-1, which encoding isoform X2 of Oxr1 (oxidation resistance 1). Oxr1 is found in plants and most other eukaryotes (from yeast to human), which is evolutionally conserved (Elliott and Volkert, 2004). Oxr1 plays an antioxidant role in eukaryotic organisms, and it was reported recently to be essential for protection against oxidative stress (Oliver et al., 2011). Considering the special antioxidation effect, the dendrobium polysaccharide is widely used in health food, pharmaccutical, chemical, and other industries as raw materials or ingredients. However, further research is needed to confirm the roles of this gene in dendrobium plants.

Another gene is of particular interest in QTL STPC-4, namely, *Dendrobium_GLEAN_10074377*, which encoding NEP1-interacting protein 2-like protein. Nep1-like proteins (NLPs) are secreted by a wide range of plant-associated microorganisms (Oome et al., 2014). Plant NLPs may be associated with polysaccharide synthesis or metabolism process. The root of *Dendrobium* species is well-known as a mycorrhizal symbiosis system, and it is hard to say this gene doesn't work in the mycorrhizal symbiosis process. In the other hand, all the experimental plants were grown in the greenhouse, and they couldn't get rid of the stress from microbes. The responses to the stress can affect the accumulation of STPC to some extent.

Of four SNPs in QTL STPC-5, three were annotated in two genes *Dendrobium_GLEAN_10120125* and *Dendrobium_GLEAN_10047849*, respectively. *Dendrobium_GLEAN_10120125* also be reported in the former study (Shen et al., 2017), which is presumably related to the polysaccharides metabolism. This gene was just one of 280 differentially expressed glycosyltransferase genes from transcriptome database, and further experimental proof is required for its potential function. The other gene *Dendrobium_GLEAN_10047849* is probable receptor-like protein kinase.

Polysaccharides are high-molecular compound, can be made up from glucose, mannose, rhamnose, xylose, and arabinose, so the metabolism of polysaccharides must be affected with too many genes. The former studies of transcriptome databases of different materials indicated the complex network of this compound (Zhang J.X. et al., 2016; Shen et al., 2017). In our study, we first focus on the STPC trait in order to detect the coverage of high density maps. On the other hand, we want to explore the genetic background complexity of STPC. At last, 5 STPC associated QTLs were detected with 19 candidate SNPs, and 8 of them were located in 4 genes. Due to lack of high quality reference genome and STPC as a chemical mixture, some STPC associated QTLs may be missed, and some candidate SNPs can't be annotated in this study. The results in this study are likely to be validated and analyzed deeply in the subsequent studies when an ideal reference genome and methods for high precision separation and determination of *Dendrobium* polysaccharides are available in the future.

In summary, the first SNP based high-density genetic linkage map constructed in this study provides an important framework for subsequent QTL mapping and MAS for *Dendrobium* species. In the long term, the STPC related QTLs detected in the present study will benefit candidate genes cloning and functional identification for *Dendrobium* polysaccharides synthesis and metabolism. In addition, two draft genome sequences of *D. officinale* have been reported (Yan et al., 2015; Zhang G.Q. et al., 2016). These genome assemblies are highly fragmented, seriously complicating correct interpretation of the *Dendrobium* genome. The genetic map of the *D. officinale* and *D. moniliforme* hybrid constructed in the present study will complement the lack of a high-density genetic linkage map for genome sequence assembly and chromosome architecture.

## Author contributions

JL and HW: Conceived the experiments, participated in the analysis and drafted the manuscript; YL and YM: Carried out the SLAF library construction and SNP genotyping; JX and ZM: Participated in the determination of total polysaccharides in F_1_ population; YS, SF, and PL: Constructed the F_1_ population. JH, CS, and XW: Performed the statistical analysis and the QTL mapping. All the authors approved the final manuscript.

### Conflict of interest statement

The authors declare that the research was conducted in the absence of any commercial or financial relationships that could be construed as a potential conflict of interest. The reviewer FC declared a shared affiliation, with no collaboration, with one of the authors, JH, to the handling Editor.

## Supplementary material

Supplementary Figure 1 Number and coverage of SLAFs for two parent and F_1_ individuals. The X-axes indicate individual accessions, including the female and male parents and followed by 111 F_1_ progenies in both **(A,B)**. The Y-axes indicate number of SLAFs in **(A)** and coverage in **(B)**.

Supplementary Figure 2 Phenotype analysis of F_1_ population.

Supplementary Figure 3 QTL for the stem total polysaccharides contents (STPC) in LG2. The x-axis scales genetic distance of dendrobe LGs accordingly, while the y-axis represents the LOD scores. The blue line represent the LOD score of every marker in LG.

Supplementary Figure 4 QTL for the stem total polysaccharides contents (STPC) in LG11. The x-axis scales genetic distance of dendrobe LGs accordingly, while the y-axis represents the LOD scores. The blue line represent the LOD score of every marker in LG.

Supplementary Figure 5 QTL for the stem total polysaccharides contents (STPC) in LG15. The x-axis scales genetic distance of dendrobe LGs accordingly, while the y-axis represents the LOD scores. The blue line represent the LOD score of every marker in LG.

Supplementary Table 1 List of segregation data of 8,573 SLAF markers.

Supplementary Table 2 Marker information and their genetic positions on each linkage group.

Supplementary Table 3 Summary of male and female linkage maps of *Dendroboium*.

Supplementary Table 4 Trait of STPC for the QTL mapping population in *Dendrobium*.

Supplementary Table 5 List of candidate genes and annotation of the STPC related QTL.
