# Supplementary figures and images for: High-Density Genetic Map Construction and Stem Total Polysaccharide Content-Related QTL Exploration for Chinese Endemic Dendrobium (Orchidaceae)
Source: Front Plant Sci. 2018 Mar 27;9:398. doi: 10.3389/fpls.2018.00398 (PMC5880926; doi:10.3389/fpls.2018.00398)

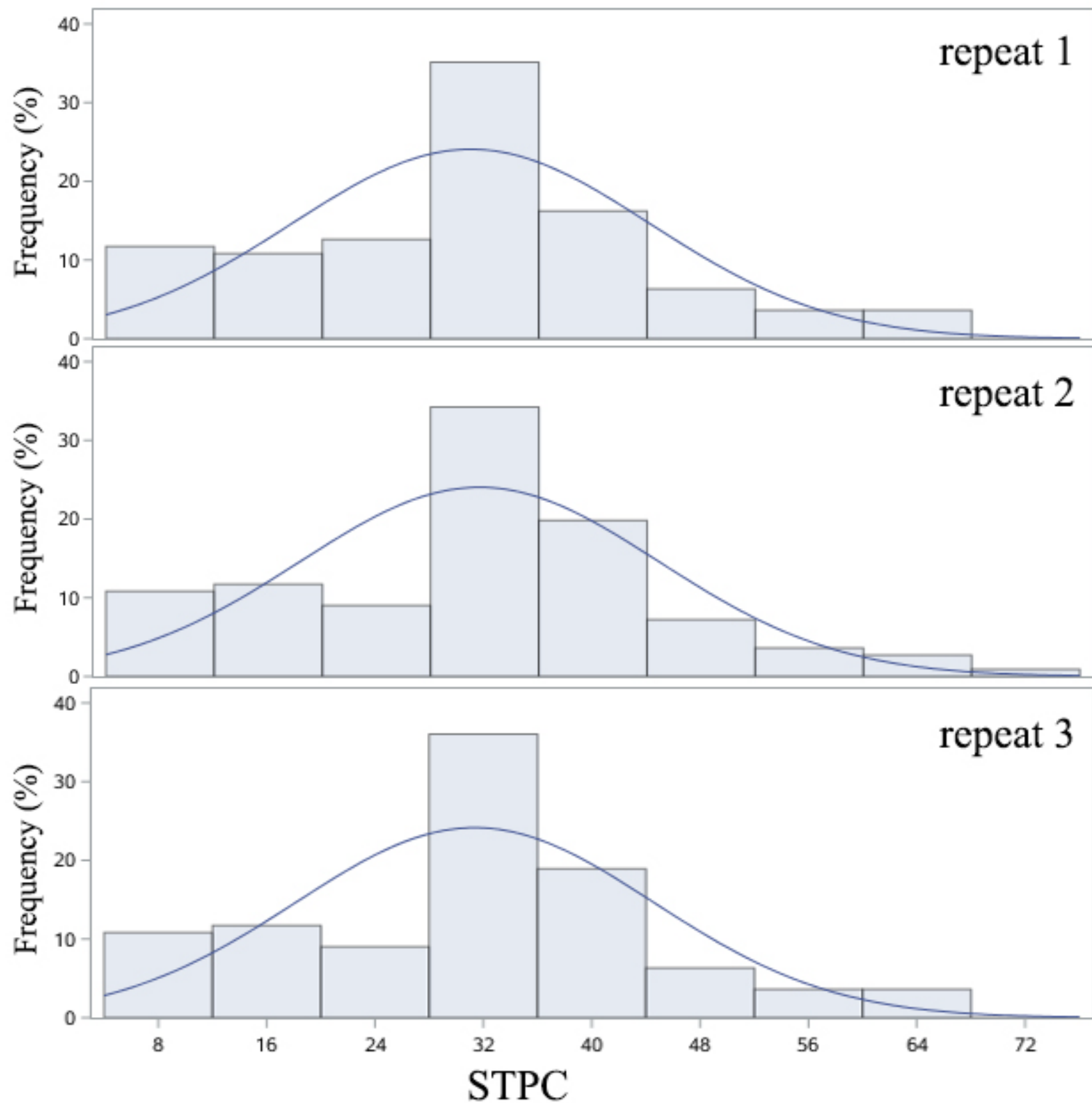

| Source | DF  | Sum of Squares | Mean Square | F Value | Pr>F   |
|--------|-----|----------------|-------------|---------|--------|
| repeat | 2   | 23.02          | 11.51       | 33.59   | <.0001 |
| line   | 110 | 57876          | 526.14      | 1535.12 | <.0001 |
| Error  | 220 | 75.40          | 0.34        |         |        |
| Total  | 332 | 57974.42       |             |         |        |

Supplement: Supplementary Figure 2 — Phenotype analysis of F1 population. [file Image2.PDF]

# STPC related QTLs on LG2

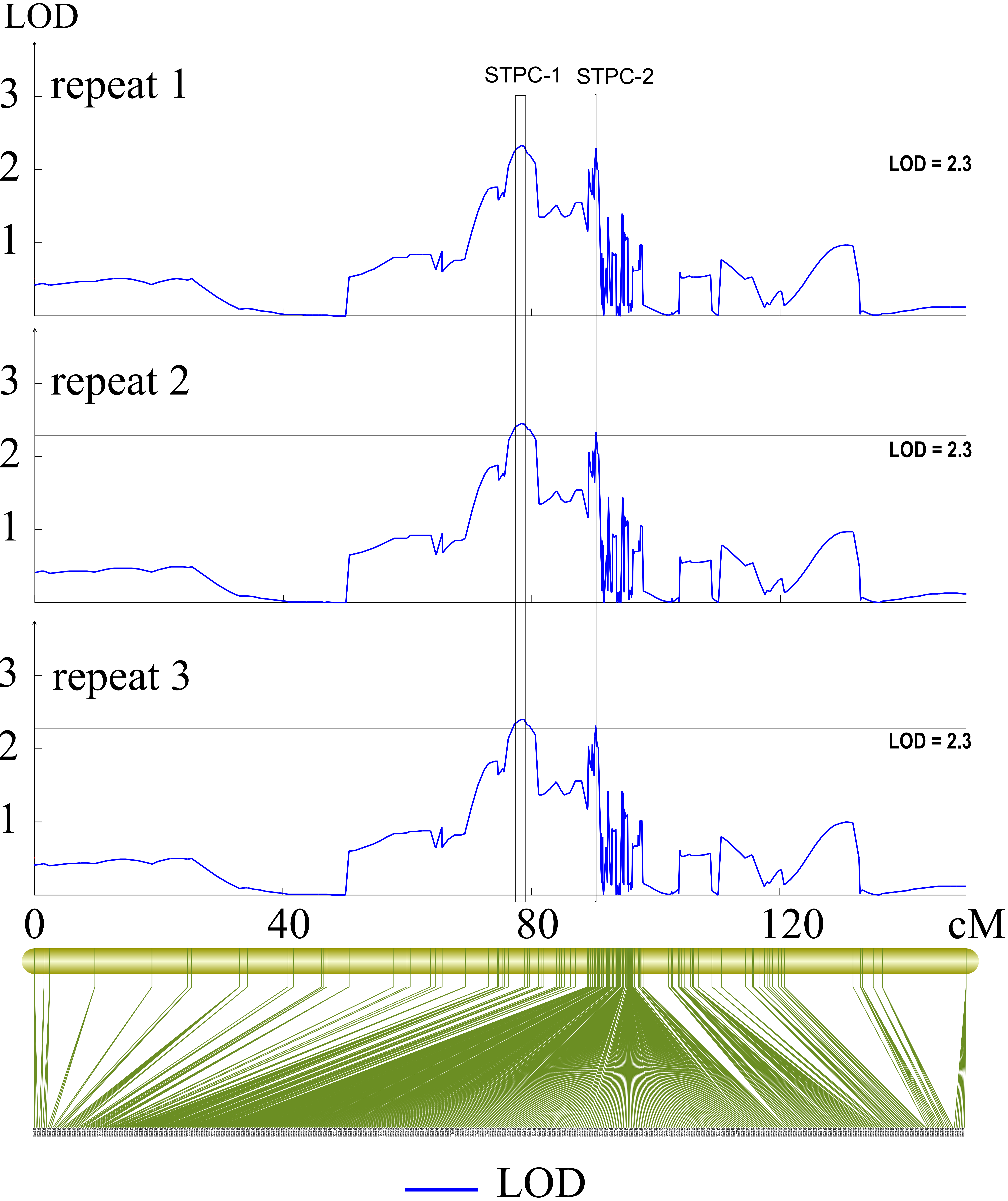

Supplement: Supplementary Figure 3 — QTL for the stem total polysaccharides contents (STPC) in LG2. The x-axis scales genetic distance of dendrobe LGs accordingly, while the y-axis represents the LOD scores. The blue line represent the LOD score of every marker in LG. [file Image3.PDF]

# STPC related QTLs on LG11

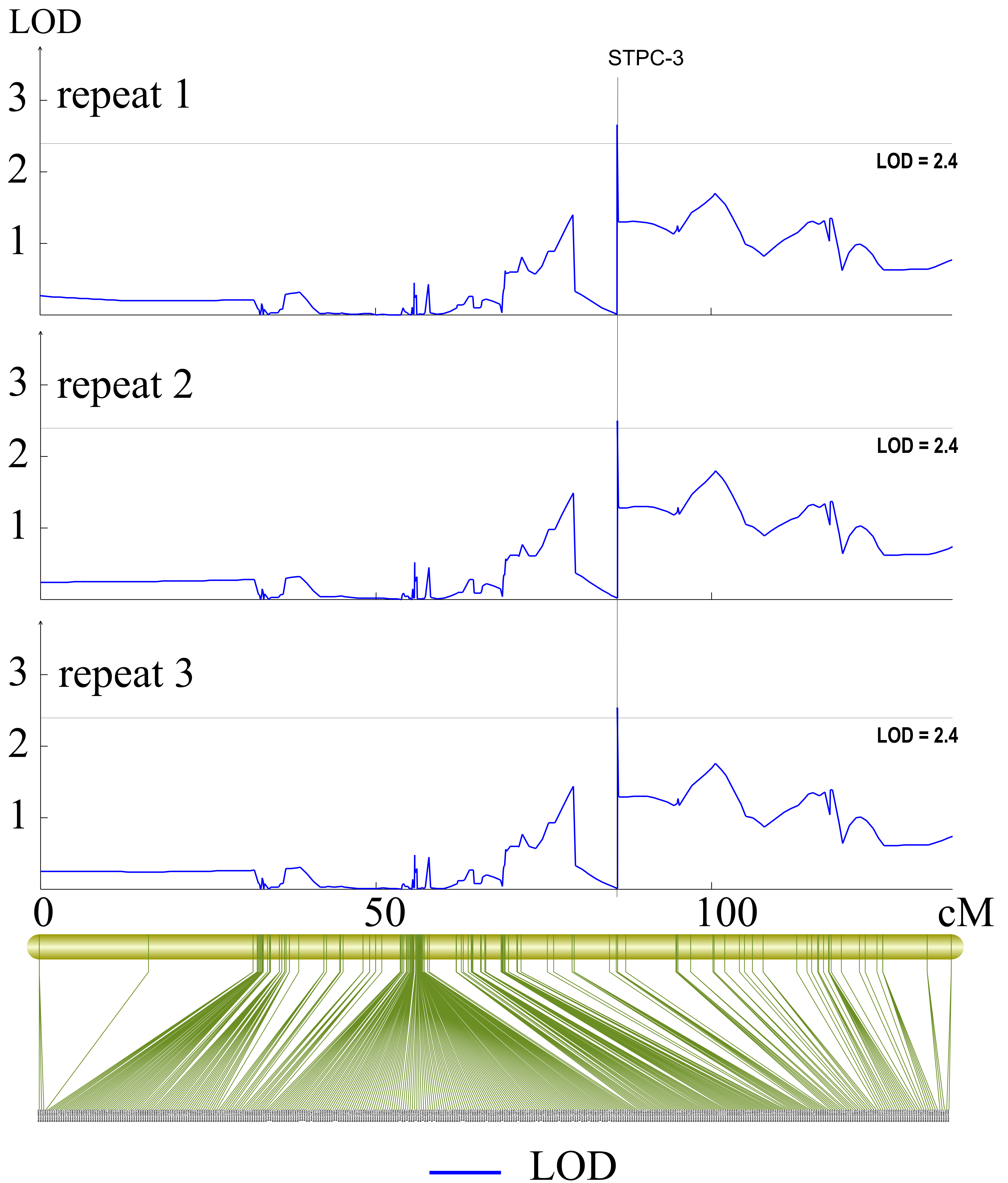

Supplement: Supplementary Figure 4 — QTL for the stem total polysaccharides contents (STPC) in LG11. The x-axis scales genetic distance of dendrobe LGs accordingly, while the y-axis represents the LOD scores. The blue line represent the LOD score of every marker in LG. [file Image4.PDF]

# STPC related QTLs on LG15

LOD

repeat 1

STPC-4 STPC-5 LOD = 2.3

repeat 2

LOD = 2.3

repeat 3

LOD = 2.3

0 50 100 cM

— LOD

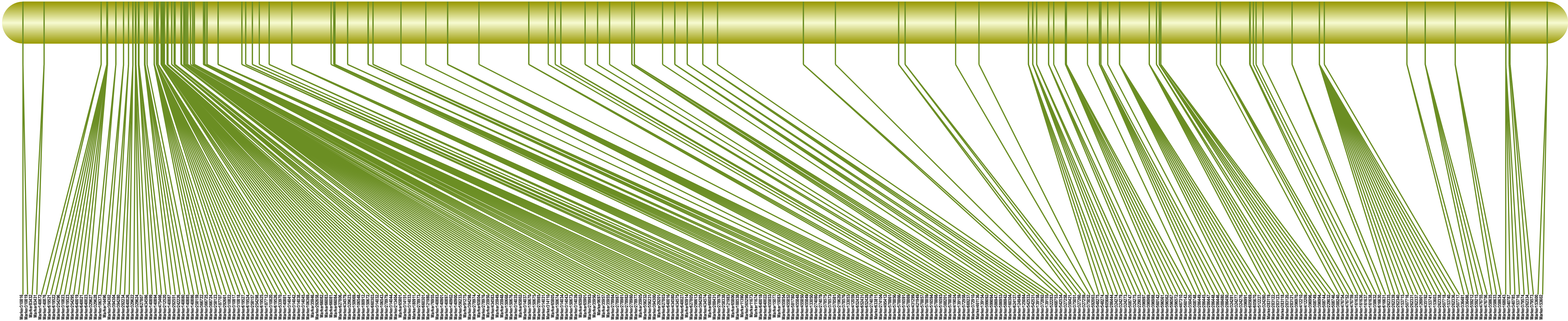

Supplement: Supplementary Figure 5 — QTL for the stem total polysaccharides contents (STPC) in LG15. The x-axis scales genetic distance of dendrobe LGs accordingly, while the y-axis represents the LOD scores. The blue line represent the LOD score of every marker in LG. [file Image5.PDF]
