# Supplementary material for: High-Density Genetic Map Construction and Stem Total Polysaccharide Content-Related QTL Exploration for Chinese Endemic Dendrobium (Orchidaceae)
Source: Front Plant Sci. 2018 Mar 27;9:398. doi: 10.3389/fpls.2018.00398 (PMC5880926; doi:10.3389/fpls.2018.00398)
Supplement: Supplementary Figure 1 — Number and coverage of SLAFs for two parent and F1 individuals. The X-axes indicate individual accessions, including the female and male parents and followed by 111 F1 progenies in both (A,B). The Y-axes indicate number of SLAFs in (A) and coverage in (B). [file Image1.PDF]

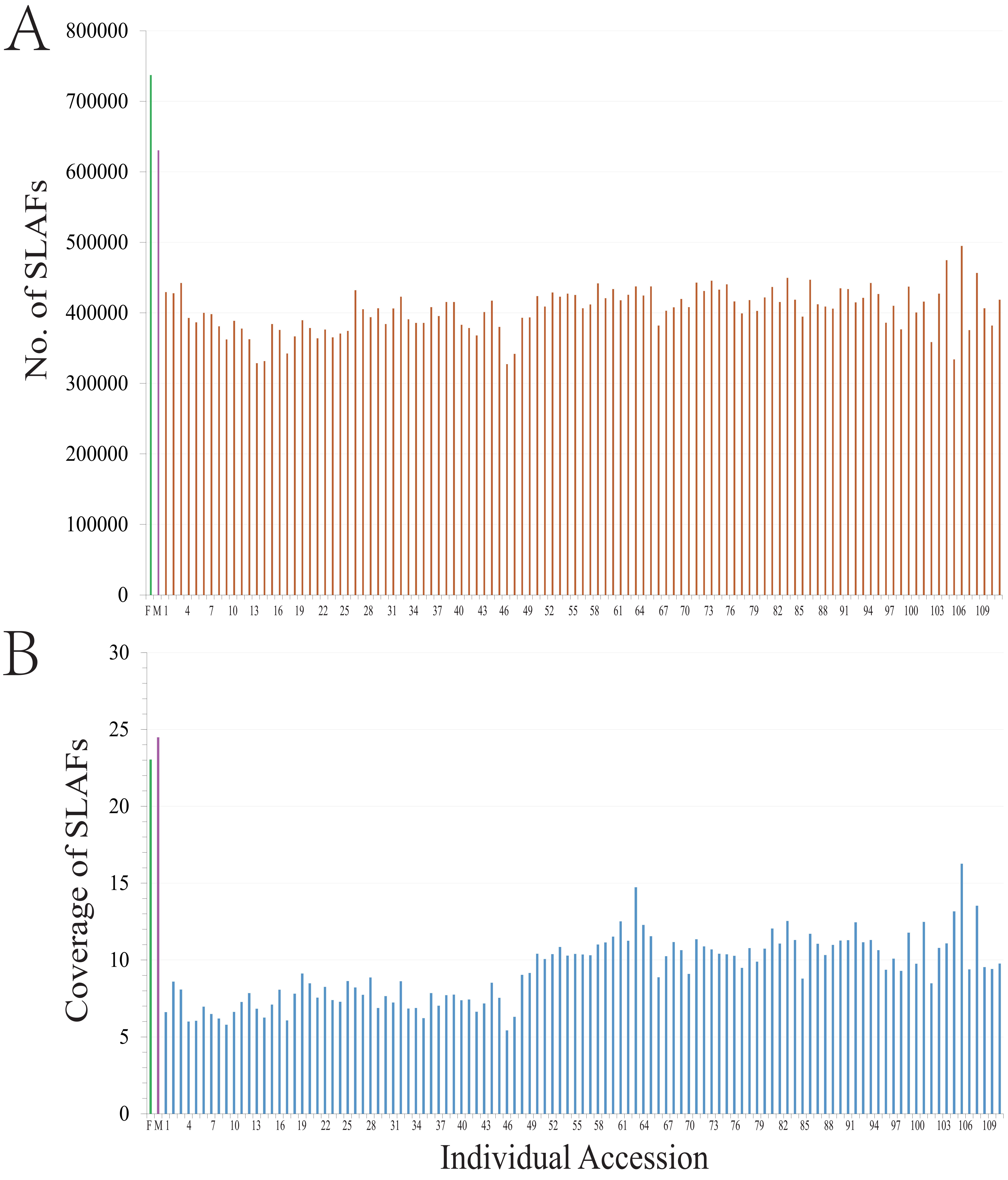
A
No. of SLAFs
800000
700000
600000
500000
400000
300000
200000
100000
0
F M 1 4 7 10 13 16 19 22 25 28 31 34 37 40 43 46 49 52 55 58 61 64 67 70 73 76 79 82 85 88 91 94 97 100 103 106 109
B
Coverage of SLAFs
30
25
20
15
10
5
0
F M 1 4 7 10 13 16 19 22 25 28 31 34 37 40 43 46 49 52 55 58 61 64 67 70 73 76 79 82 85 88 91 94 97 100 103 106 109
Individual Accession
